# Part Two: Evaluation of *N-*methylbupropion as a Potential Bupropion Prodrug

**DOI:** 10.3390/ph7060676

**Published:** 2014-05-28

**Authors:** Paul Matthew O’Byrne, Robert williams, John J. walsh, John F. Gilmer

**Affiliations:** School of Pharmacy and Pharmaceutical Sciences, University of Dublin, Trinity College, Dublin 9, Ireland; E-Mails: robert.williams@lifescientific.com (R.W.); jjwalsh@tcd.ie (J.J.W.); gilmerjf@tcd.ie (J.F.G.)

**Keywords:** *N-*methylbupropion, bupropion, bioprecursor, prodrug, microsomes, plasma, *in vivo*, metabolism

## Abstract

*N-*methylbupropion was selected as a potential prodrug from our *in vitro* screening of analogues of bupropion described in the preceding paper. This study describes *in vivo* pharmacokinetics of *N-*methylbupropion in the guinea-pig animal model, which is reported to best predict human metabolism of bupropion. The suitability of the guinea pig was established by studying *N-*demethylation of *N-*methylbupropion using S9 liver fractions. An LC-MS method was developed and validated to measure *N-*methylbupropion, bupropion and their metabolites in plasma and brain tissue. In separate studies, the prodrug was delivered by intraperitoneal injection (IP) to assess hepatic metabolism and then by oral gavage (PO) to assess the contribution from intestinal enzymes. Bupropion was administered in parallel. The pharmacokinetic profile of bupropion and *N*-methylbupropion were not comparable when dosed by intraperitoneal injection but when dosed orally, *N-*methylbupropion showed a comparable bupropion and metabolite PK plasma profile to bupropion. Plasma and brain levels of *N-*methylbupropion show that it is extensively metabolized to bupropion and its metabolites, and *N-*methyl-*threo*-hydrobupropion. This data coupled to the reduced DAT and NET system *in vitro* activity described in paper 1 would suggest that the *N-*methyl derivative of bupropion may have potential as an oral prodrug of bupropion in humans.

## 1. Introduction

Bupropion is an α-aminoketone that acts primarily at four therapeutic sites, affecting dopamine, norepinephrine, and nicotinic acetylcholine activity and by modulating cytokine levels including tumor necrosis factor-α and interferon*-*γ. Its therapeutic uses range from CNS diseases such as depression [1] to systemic inflammatory disease. Numerous studies have been carried out to examine the effectiveness of bupropion in a tobacco-use cessation therapy [2,3], in Crohn’s disease [4,5,6,7,8,9,10,11,12,13,14], pain [15,16,17], attention deficit hyperactivity disorder [18], restless leg syndrome [19] and seasonal affective disorder [20,21] as well as an adjuvant treatment for multiple myeloma [8,11]. For the twelve months ending 30 June 2013, Bupropion Hydrochloride Extended-release Tablets, 150 mg and 300 mg, had U.S. sales of approximately $503.3 million, according to IMS Health.

Bupropion is generally well tolerated. The most common adverse effects during initial treatment are dry mouth, constipation, headache, nausea, agitation, insomnia and weight loss [22]. These side effects are common for drugs that work on the noradrenergic and dopaminergic functions. The most common cause for stopping treatment is jitteriness or an unpleasant state. The most contentious side effect is seizure liability and in 1986, shortly after its approval by the US FDA, bupropion was withdrawn. It was reintroduced in 1989 at a lower dose range but currently has contraindications for use in patients with seizure history, eating disorders or those undergoing ethanol or other CNS depressant withdrawal [23]. There is evidence that the adverse effects associated with treatment are due to the metabolites [24]. Bupropion freebase is also susceptible to stability issues and suffers from hydrolysis and oxidation at pH > 5 [25].

**Scheme 1:**
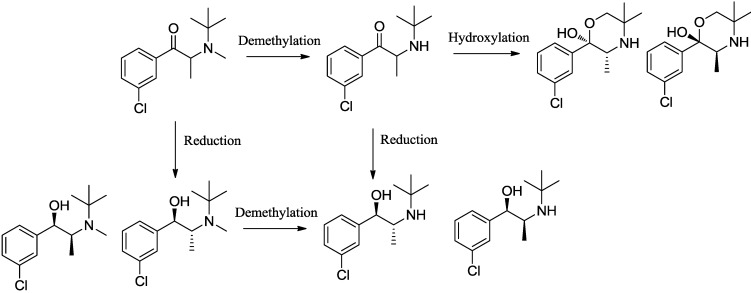
*N*-methylbupropion, its metabolite, bupropion and its active metabolites as reported in paper 1 of this two part series.

The metabolic and pharmacokinetic profile of bupropion is scientifically interesting and clinically significant because the metabolites contribute to the pharmacological [26] and side effect profile of bupropion [24]. Bupropion is extensively metabolized in the liver and intestine producing three basic active metabolites, hydroxybupropion, *threo*-hydrobupropion and *erythro-*hydrobupropion (Scheme 1). Plasma levels of the metabolites are several times higher than bupropion soon after oral dosing in man.

The pharmacokinetics of bupropion were studied in several species [27,28,29] including rat, mouse and guinea pig. The species that best represents human metabolism is the guinea pig. The rat and mouse do not produce significant amounts of the reduced metabolite *threo*-hydrobupropion which is a significant metabolic product of human metabolism. *erythro*-Hydrobupropion is not a prominent metabolite in animal models. Work on potential co-drugs of bupropion [30] and prodrugs of hydroxybupropion [31] was also carried out in the guinea pig.

In the preceding paper of this two part series we described a systematic approach leading to the identification of *N-*alkyl derivatives as potential prodrugs of bupropion. The *N-*methyl derivative of bupropion was selected as a lead candidate for further *in vivo* studies due to its lower activity at the dopamine active transport (DAT) and norepinephrine transport (NET) systems compared to bupropion.

The potential use of *N-*methylbupropion as a pharmacotherapy for cocaine addiction has been suggested [32]. Another study suggested *N-*methylbupropion as a possible aid to smoking cessation for [33]. Although this compound may have some activity in specific *in vitro* assays, its use is limited as an orally delivered drug due to its extensive metabolism to bupropion as shown herein.

In this paper we describe the development and validation of an analytical method to determine the concentrations of *N-*methylbupropion, bupropion and their metabolites in the guinea pig for a pharmacokinetic study. We then evaluated the metabolism of *N-*methylbupropion and bupropion following oral and IP dosing in the guinea pig. The *N-*methyl derivative emerges as a potential prodrug in the guinea pig, vindicating the selection process described in our earlier paper. Interesting differences emerged between the profiles generated from the two routes due to the contributions of intestinal enzymes.

## 2. Experimental Section

### 2.1. Instrumentation

Chromatographic analysis was carried out on a Thermo Accela liquid chromatograph (Thermo Fisher Scientific, Waltham, MA, USA). The detector was a Thermo LTQ-XL-Orbitrap Discovery mass spectrometer. Centrifugation was carried out on a Micromax centrifuge (Thermo Fisher Scientific). Vortex mixing was carried out on a Rx3 vortex mixer (Velp Scientifica, Usmate, Italy). Standards were stored in a Thermo Forma −86 °C ULT freezer. Brain samples were homogenized using a T10 basic homogenizer (IKA, Staufen, Germany).

### 2.2. Materials

Bupropion hydrobromide reference standard was supplied from Biovail Technologies Ireland Ltd. (Dublin, Ireland) Guinea pig S9 fraction, and NADP regenerating solutions were supplied by BDBiosciences (Franklin Lakes, NJ, USA). LC-MS grade solvents were purchased from Fisher Scientific Ireland (Dublin, Ireland). HPLC grade solvents and all other reagents were analytical grade and purchased from Sigma Aldrich (St. Louis, MO, USA). Bupropion hydrochloride reference standard and its metabolite standards were purchased from Toronto Research Chemicals (North York, ON, Canada). *N-*methylbupropion amino alcohol metabolites, *Rac*-*N-*methyl-*erythro*-hydrobupropion and *Rac*-*N-*methyl-*threo*-hydrobupropion were synthesized and characterized at Trinity College Dublin. All stock standards and solutions were stored at −80 °C and sub-aliquoted to reduce freeze thaw cycles. Ammonium hydroxide solution (30% as NH_3_) HPLC grade, formic acid HPLC grade, methanol, water and acetonitrile all LC-MS grade were obtained from Fischer Scientific.

### 2.3. Synthesis of Rac-N-methyl-erythro-hydrobupropion and Rac-N-methyl-threo-hydrobupropion (Amino Alcohol Metabolites of N-Methylbupropion)

To *N-*methylbupropion (0.05 g, 0.2 mmol) was added sodium borohydride (0.04 g, 1.0 mmol) in methanol-ethanol (1:4, 5 mL) at room temperature. The mixture was stirred for 30 min. The color changed from slight yellow to clear cloudy. The reaction was complete when no *N-*methylbupropion was detected by thin layer chromatographic analysis. Water (10 mL) was added and the mixture was extracted with hexane (3 × 10 mL portions). The hexane extracts were combined and dried over anhydrous magnesium sulfate. The hexane was removed under vacuum on a rotary evaporator. A white solid of the two diastereomers remained after drying. Yield 0.05 g (100%, 66% *erythro*, 33% *threo* by NMR and LCMS).

*N-*Methyl-*erythro*-aminoalcohol. ^1^H-NMR δ (CDCl_3_): 0.96–0.98 (d, 3H, *J =* 7.0 Hz, -CHCH_3_), 1.10 (s, 9H, -CH(CH_3_)_3_), 1.99 (s, 3H, -NCH_3_), 3.36–3.43 (q, 1H, *J =* 6.7 Hz, -CHCH_3_), 4.48–4.49 (d, 1H, *J =* 5.36 Hz, -CHOH), 7.19–7.39 (m, 4H, Ar-H). ^13^C-NMR ppm: 12.72 -CHCH_3_, 26.7 -CH(CH_3_)_3_, 30.5 -NCH_3_, 54.5 -CHCH_3_, 54.5 -C(CH_3_)_3_, 73.9 -CHOH, 124.6, 126.4, 126.5, 128.3 (4 × Ar-C), 133.2, 144.7 (2 × Ar-C). HRMS (M+H) actual 256.1463 found 256.1460.

*N-*Methyl-*threo*-aminoalcohol. ^1^H-NMR δ (CDCl_3_): 0.88–0.90 (d, 3H, *J =* 6.41 Hz, -CHCH_3_), 1.19 (s, 9H, -CH(CH_3_)_3_), 2.25 (s, 3H, NCH_3_), 2.95–3.01 (m, 1H, -CHCH_3_), 4.02–4.04 (d, 1H, *J =* 5.36 Hz, -CHOH), 7.19–7.39 (m, 4H, Ar-H). ^13^C-NMR ppm: 11.72 -CHCH_3_, 27.3 -CH(CH_3_)_3_, 27.3 -NCH_3_, 57.7 -CHCH_3_, 55.0 -C(CH_3_)_3_, 73.5 -CHOH, 125.3, 126.9, 127.2, 128.9 (4 × Ar-C), 133.7, 144.9 (2 × Ar-C). HRMS (M+H) actual 256.1463 found 256.1455.

### 2.4. Chromatographic Method for the Determination of N-Methylbupropion, Bupropion and Metabolites

The column used for chromatographic separation was a Waters Xbridge C18, 2.1 × 50 mm 2.5 µm at 30 °C. Mobile phase A: 10:40:50, 1.0% ammonium hydroxide solution in water, adjusted to pH 10.5 with formic acid: water: ACN. Mobile phase B: 10:90, 1.0% ammonium hydroxide solution in water, adjusted to pH 10.5 with formic acid: ACN. Flow rate: 250 µL/min, injection volume: 10 µL, run time: 10 min. Gradient, 100% A hold for 1 min, 0% B to 100% B over 4 min, hold 100% B for 4.5 min, 0% B at 9.51 min and equilibrate for 0.5 min.

### 2.5. Mass Spectrometer Conditions for Detection N-Methylbupropion, Bupropion and Metabolites

The LTQ-XL ion trap mass spectrometer was coupled to the Accela LC system via an electrospray ionization (ESI) probe. The capillary temperature was maintained at 350 °C, sheath gas flow rate 50 arbitrary units, auxiliary gas flow rate 5 arbitrary units, sweep gas flow rate 0 arbitrary units, source voltage 3.20 kV, source current 100 µA, capillary voltage 43.00 V and tube lens 100 V. Compounds were detected in positive ion mode using selected ion monitoring (SIM). Hydroxybupropion was detected at (M+H)^+^ = 256.1099, RT = 1.0 min, *Rac*-*erythro*-hydrobupropion (M+H)^+^ = 242.1306, RT = 2.3 min, *Rac-threo*-hydrobupropion (M+H)^+^ = 242.1306, RT 2.8 min, bupropion (M+H)^+^ = 240.1150, RT = 2.0 min, *N-*methylbupropion (M+H)^+^ = 254.1306, RT = 4.9 min, *Rac*-*N-*methyl-*erythro*-hydrobupropion (M+H)^+^ = 256.1463, RT = 4.1 min and *Rac*-*N-*methyl-*threo*-hydrobupropion (M+H)^+^ = 256.1463, RT = 4.6 min. The optimum detector conditions were found by tuning the instrument to be most sensitive for bupropion’s most abundant ion at 240 (*m/z*).

### 2.6. N-Demethylation of N-Methyl Bupropion to Bupropion by Guinea Pig S9 Liver Fraction

Guinea pig S9 liver fraction, NADPH regenerating solutions A and B were thawed rapidly to 37 °C, then kept on wet ice until ready for use. A number of different concentration substrate solutions were prepared were the maximum concentration of DMSO was 1%, and the concentration of guinea pig S9 liver fraction, NADP regenerating system solution B and phosphate buffer was fixed. After incubation for 5 min, NADP regenerating system solution A was added and the metabolism was initiated. Final volume was 100 μL. The tube was inverted twice and vortex mixed. The final concentrations in this solution was 50–1000 μM prodrug, 0.1 mg/mL guinea-pig S9 liver fraction, 1.3 mM NADP^+^, 0.4 U/mL glucose-6-phosphate dehydrogenase and 1.0% DMSO. This solution was incubated for 20 min at 37 °C. The reaction was quenched by addition of 100 μL of ACN. The mixture was centrifuged at 10,000 × *g* for 10 min. The supernatant was analysed by LC-MS analysis.

### 2.7. Standard Preparation

Stock standard solutions of bupropion free base were prepared at 10 mg/mL in MeOH (42 mM), for example 9.0 mg bupropion hydrochloride was dissolved in MeOH (779 µL) (42 mM). Stock standard solutions were prepared in duplicate and verified off each other at working standard level to ensure accurate preparation. Standard verification involves checking the area response to concentration ratio of each replicate standard, and calculating the% difference between the preparations. It is a measure of the accuracy and reproducibility of standard preparation. Acceptable standard verification was 95%–105%. Working standard solutions were prepared by serial dilution of stock standards in 0.001 M HCl.

### 2.8. System Suitability

System suitability of the LC-MS system was established on the day of use by repeated injection of a working standard solution. The% RSD of six replicate standard injections for retention time and area was not more than 1.0%. This ensured that the system was stable, equilibrated and suitable for analysis. System suitability throughout the analysis run and at the end of the analysis run was also established by performing a quality control standard injection. This ensured system drift was kept to a minimum. The quality control sample was injected after every eight samples. The% RSD of the quality control samples was less than 5%.

### 2.9. In Vivo Pharmacokinetic Studies

The study plans of both the intraperitoneal and oral dosing animal studies were approved by the ethics departments of Charles Rivers Laboratories and MDS Pharma Services Laboratories prior to execution of the studies at their GLP accredited sites.

#### 2.9.1. Intraperitoneal and Oral Animal Studies

After acclimatization, male Hartley albino guinea-pigs, weighing 280–320 g were deprived of food for 16 h with free access to water (food withdrawal at 04:00 PM the day before). On the day of the experiment five guinea pigs per group were weighed then injected through the abdominal wall or fed by oral gavage with bupropion given at 40 mg/kg intraperitoneum. A second group of five guinea pigs were weighed then injected through the abdominal wall or fed by oral gavage with *N-*methyl bupropion given at 42 mg/Kg intraperitoneum.

Guinea pigs were placed under isoflurane anesthesia and approximately 100 µL of blood was collected using hematocrit capillaries or by cardiac puncture (terminal) into dry heparin/Li containing tubes before administration and then at five time points post-administration (0, 20, 40, 60, 120 and 180 min) for intraperitoneal study and seven time-points post administration (0, 20, 40, 60, 120, 180, 240 and 360 min) for oral study. After blood was collected, the tubes were inverted several times and placed on ice. The samples were centrifuged within 30 min maximum after sampling at 2205 g at 4 °C for 10 min. Plasma was transferred to polypropylene tubes and frozen with minimal delay at approximately −70 °C.

An additional group of guinea-pigs were used for brain collection at a single time point (20 min). The day of the experiment four guinea pigs per group were weighed then injected through the abdominal wall with bupropion given at 40 mg/kg IP and *N-*methylbupropion given at 42 mg/Kg IP. At time 20 min post-injection, the animals were decapitated and the brain extracted and rinsed in cold physiological saline. The whole brain was fast frozen on dry ice and placed into pre-labeled vials and stored at approximately −70 °C (deep frozen).>

#### 2.9.2. Plasma Sample Preparation

Guinea pig plasma was defrosted and centrifuged. Then, plasma (50 µL) was added to a 1.5 mL microcentrifuge tube. ACN (200 µL) was added and the mixture was vortex mixed. The resulting solution was centrifuged at 8000 *g* for 10 min. Supernatant (100 µL) was added to HCl (100 µL, 0.001 M). The solution was vortex mixed and added to a 100 µL micro-insert for LC-MS analysis. 

#### 2.9.3. Brain Sample Preparation

Guinea pig brain was defrosted. The whole brain was weighed into a beaker and 0.01 M HCl (20 mL) was added. The mixture was homogenized for 3 min on level 6 and allowed to settle for 10 min. An aliquot of the resulting suspension was centrifuged at 10,000 *g* for 10 min. Supernatant (250 µL) was added to ACN 750 µL. The mixture was centrifuged at 10,000 *g* for 10 min. 200 µL of the supernatant was added to 800 µL water. The clear solution was vortex mixed and analyzed by LC-MS.

#### 2.9.4. Method Validation

The experimental of the method validation can be found in supplemental information.

#### 2.9.5. Pharmacokinetic Measurements

Pharmacokinetic calculations were determined on Graphpad Prism software. Michaelis-Menten parameters were determined by non*-*linear regression analysis. The pharmacokinetic data was interpreted using a one-compartment model where the amount of drug present in the body at any given time t [*A(t)*] is described in Equation (1).

Drug concentration:

*A*(*t*) = *C_p_*(*t*)*V* (1)

where *Cp*(t) and *V* are the drug concentration in the plasma and the apparent volume of distribution respectively. At least five sample time points were collected including at least one time point before *t_max_* and three time points during the terminal phase for half-life estimation. AUC, *C_max_* and *t_max_* were estimated directly from PK data but terminal half-life was calculated by plotting the natural logarithm of concentration *vs.* time, the slope of which gave the elimination rate constant β. The terminal half-life t_½_ was determined from Equation (2);

Terminal half-life:

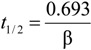
(2)


#### 2.9.6. Statistical Analysis

*C_max_* values between bupropion and *N-*methylbupropion PK studies were analysed for their similarity using an Unpaired *t*-test. A 90% confidence interval was used as stated in the EU guidelines of medicinal pharmaceutical-equivalence studies. All statistical analyses were performed using Prism version 4 for Windows, GraphPad Software (La Jolla, CA, USA).

## 3. Results and Discussion

### 3.1. Method Development

The chromatographic method was developed initially by running four different scouting gradients over two different time intervals. The standard sample mixture consisted of bupropion and its three metabolites, hydroxybupropion, *threo*-hydrobupropion and *erythro*-hydrobupropion as well as *N-*methylbupropion and its two potential *in vivo* metabolites *N-*methyl-*threo*-hydrobupropion and *N-*methyl-*erythro*-hydrobupropion. Two aqueous buffers were evaluated for the mobile phase, a pH 2.5 ammonium formate and pH 10.0 ammonium formate buffer. Two different organic solvents were evaluated as organic modifiers, ACN and MeOH. Linear gradients from 10% B to 90% B over 10 min and 30 min were examined. Resolution, selectivity and peak shape were determined in assessing the optimum mobile phase. Working at low pH, there was inadequate retention on the RP column, due to all analytes being predominantly in their ionized form. The poor retention caused inadequate resolution between bupropion and its metabolites. High pH mobile phase gave excellent retention on the column as all analytes were predominantly unionized. ACN gave a better overall peak shape than MeOH but there was a loss of resolution between bupropion and *threo*-hydrobupropion.

**Figure 1:**
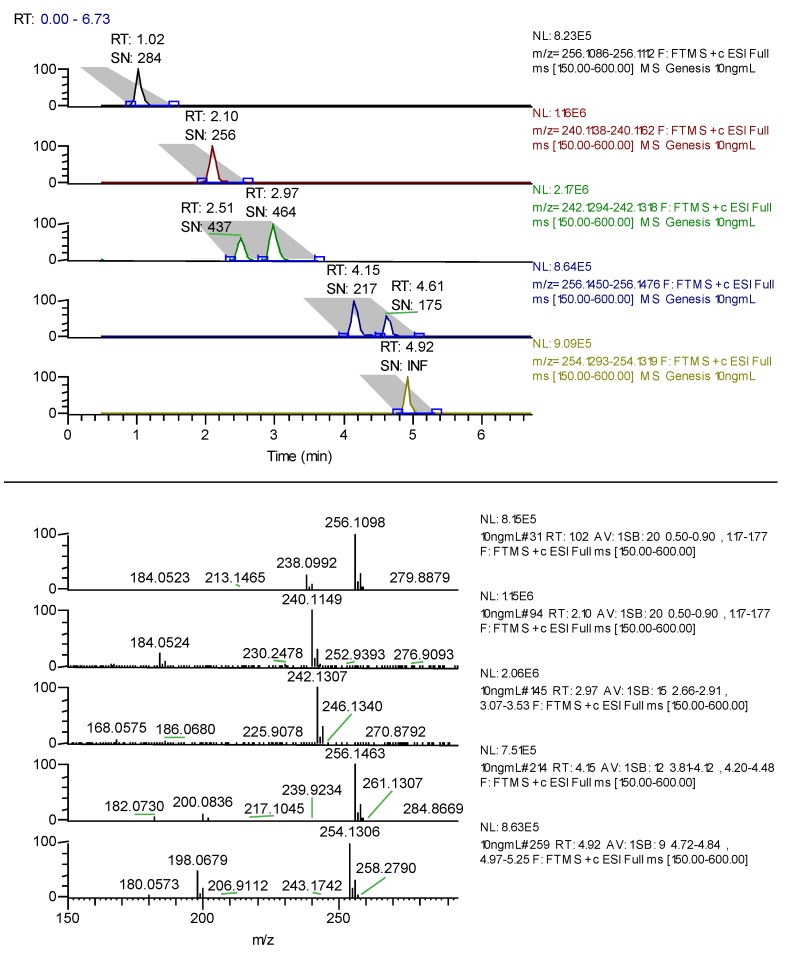
LC-FTMS chromatogram of hydroxybupropion 1.02 min, bupropion 2.1 min, *erythro*-hydrobupropion 2.51 min and *threo*-hydrobupropion 2.97 min, *N*-methyl-*erythro*-hydrobupropion 4.15 min, *N*-methyl-*threo*-hydrobupropion 4.61 min and the prodrug N-methylbupropion 4.92 min each at 10 ng/mL. Under the peak retention times is the signal to noise ratio SN.

The use of MeOH in the mobile phase gave better overall resolution at pH 10.0. Adjustment of the pH to 10.5 gave better resolution using ACN as organic modifier. The retention time of bupropion was affected considerably by changes in the pH of mobile phase but pH 10.5 gave the best resolution between all metabolites, bupropion and prodrug. The gradient was then optimized to shorten run time and improve efficiency. A sample chromatogram is presented in Figure 1.

During the optimization of the chromatographic conditions, the mass spectrometer was monitoring eluant peaks in positive ion electrospray mode. The instrument was tuned on bupropion’s (M+H^+^) peak, 240.1150 amu. Sheath gas was optimized to improve nebulization of the sample solution in the electrospray. The auxiliary gas was optimized to improve the vapor plume coming from the electrospray. This helped focus the spray to the ion source and improved desolvation. No sweep gas was used as this did not affect the sensitivity of the electrospray at any value. The capillary temperature was optimized by running the same experiments three times at capillary temperatures of 200, 300 and 400 °C. The temperature was chosen which gave the best sensitivity. The capillary voltage, tube lens and other focusing lenses were autotuned using the MS tune software.

### 3.2. Optimization of Protein Precipitation

A number of different protein precipitation techniques were evaluated to ensure full extraction of analytes. Our initial in*-*house method utilized addition of a 2% zinc sulfate solution in 50% ACN to the plasma samples, which was effective for precipitating plasma proteins. However, quenching with zinc sulfate resulted in low bupropion recovery. Plasma protein precipitation was then evaluated with ACN, MeOH and THF. ACN afforded the best recovery and reproducibility. It was also chosen as it was our organic modifier in our chromatographic method, so injection of this up to 50% composition would not adversely affect chromatography.

### 3.3. Method Validation

The method validation results can be found in the supplemental information.

### 3.4. N-Demethylation of N-Methyl Bupropion to Bupropion by Guinea Pig S9 Liver Fraction

**Figure 2:**
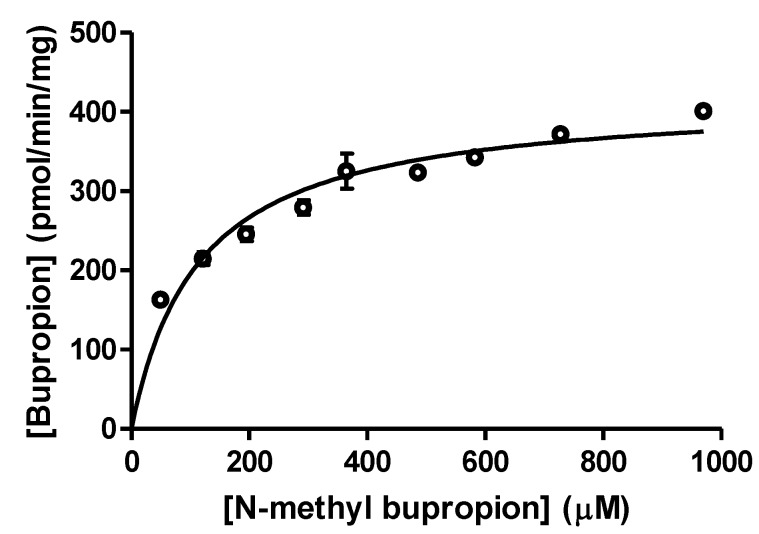
A substrate saturation curve for *N*-methylbupropion in guinea pig S9 liver fraction, V_max_ = 419.2 ± 13.65 pmol/min/mg S9 (*n =* 3).

In selecting a guinea pig animal model for an *in vivo* study, it was prudent to first verify if an *in vitro* guinea pig model would carry out the same metabolic transformations. A substrate saturation study was performed using guinea pig S9 liver fractions to verify if *N-*demethylation occurred in guinea pigs as compared with human liver microsome preparations. *N-*methylbupropion and bupropion concentrations were determined by LC-MS. Since *N-*methylbupropion demethylation occurred readily, the data demonstrated suitability of the guinea pig animal model to study the pharmacokinetics of *N-*methyl bupropion transformation to bupropion, Figure 2. Known metabolites of bupropion such as hydroxybupropion were identified in the guinea pig S9 fraction. Other unknown minor metabolites of *N-*methylbupropion were also detected but were not quantified at this stage. These were determined during the *in vivo* stage, discussed below.

### 3.5. In Vivo Study Results

### 3.6. Pharmacokinetics of N-Methylbupropion and Bupropion in Guinea Pig Following Intraperitoneal Injection

**Figure 3:**
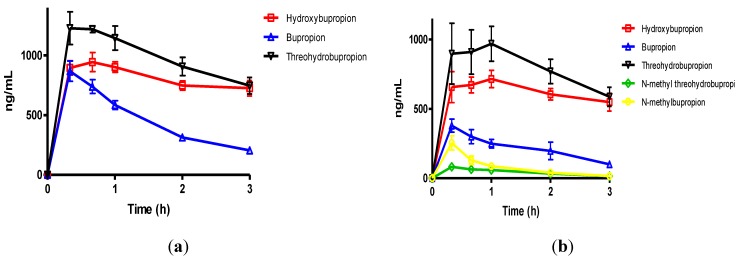
(**a**) The mean plasma levels of bupropion and metabolites after dosing 40 mg/Kg bupropion to guinea pigs by IP injection (*n* = 5); (**b**) The mean plasma concentrations of *N*-methylbupropion and metabolites after dosing 42 mg/Kg N-methylbupropion to guinea pigs by IP injection (*n* = 5).

**Figure 4:**
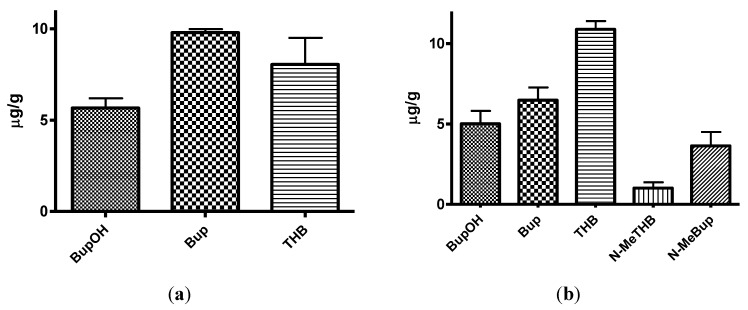
(**a**) The mean brain concentrations of bupropion and metabolites 20 min after dosing 40 mg/Kg bupropion to guinea pig by IP injection (*n* = 5); (**b**) The mean brain concentrations of *N*-methylbupropion and metabolites 20 min after dosing 42 mg/Kg *N*-methylbupropion to guinea pigs by IP injection (*n* = 5).

The IP route of administration was chosen for guinea pig administration in the present work because it is considered to be a route that involves the first-pass effect and therefore gains access to the hepatic-portal system. A 40 mg/Kg bupropion dose was chosen for the guinea pig so the data could be directly compared to literature [29] to establish the validity of our method; an equimolar amount of prodrug was dosed alongside bupropion. The pharmacokinetic data after dosing bupropion and *N*-methyl bupropion via IP injection are given in Table 1, Table 2 and Table 3. These data sets are presented graphically in Figure 3 and Figure 4.

**Table 1:** The pharmacokinetic parameters for *N*-methylbupropion, bupropion and metabolites following equimolar dosing via intraperitoneal injection in guinea pig study (*n =* 5).

Bupropion dosed at 40 mg/Kg			Parameter	N-Methylbupropion dosed at 42 mg/Kg				
BupOH	Bup	THB	Metabolite	BupOH	Bup	THB	N-Me Bup	N-Me THB
2.33	1.34	2.86	**AUC_0-180 _(µg.h/mL)**	1.80	0.64	2.31	0.23	0.13
0.95 ± 0.17	0.87 ± 0.19	1.22 ± 0.31	**Mean C_max _(µg/mL) ± SD**	0.72 ± 0.14	0.38 ± 0.09	0.97 ± 0.28	0.18 ± 0.08	0.08 ± 0.02
0.66	0.33	0.33	**t_max_ (h)**	1.00	0.33	1.0	0.33	0.33
6.8	1.2	3.3	**t½ (h)**	5.2	1.5	4.0	0.7	1.1

**SD **= standard deviation.

**Table 2:** The brain/plasma ratio of bupropion and metabolites in the guinea-pig 20 min post IP injection of 40 mg/Kg bupropion (*n =* 5).

Metabolite	Brain levels		Plasma levels		Brain/plasma ratio
	μg/g	SD	μg/mL	SD	
**BupOH**	5.67	1.06	0.90	0.13	6.30
**Bup**	9.79	0.37	0.87	0.19	11.25
**THB**	8.04	2.92	1.23	0.31	6.54

**Table 3:** The brain/plasma ratio of *N*-methylbupropion and metabolites in the guinea-pig 20 min post IP injection of 42 mg/Kg *N*-methylbupropion (*n =* 5).

Metabolite	Brain levels		Plasma levels		Brain/plasma ratio
	μg/g	SD	μg/mL	SD	
**BupOH**	5.02	1.60	0.66	0.25	7.61
**Bup**	6.49	1.56	0.38	0.09	17.07
**THB**	10.89	1.03	0.90	0.49	12.1
***N-*MeTHB**	1.01	0.72	0.08	0.02	12.63
***N-*MeBup**	3.64	1.73	0.18	0.08	20.22

After dosing bupropion 40 mg/Kg, the metabolite with the highest AUC was *threo*-hydrobupropion (THB) (2.86 μg/mL.h), followed by hydroxybupropion (BUPOH) (2.33 μg/mL.h). Bupropion (BUP) was observed at lower levels than both metabolites with an AUC of 1.34 μg/mL.h. This is the expected profile seen in the guinea pig, arising from extensive first pass hepatic metabolism. The *t_max_* for *threo-*hydrobupropion and bupropion were 20 min, and hydroxybupropion was 40 min. The *C_max_* for *threo*-hydrobupropion, hydroxybupropion and bupropion were estimated to be 1228 ± 307, 945 ± 178 and 869 ± 190 ng/mL respectively. Bupropion and metabolite levels in the brain did not show a similar profile. The brain/plasma ratios of bupropion and metabolites were calculated at 20 min. Bupropion has the highest B/P of 11.25, followed by *threo*-hydrobupropion 6.54 and hydroxybupropion 6.30. This is reflective of its lipophilicity and is consistent with literature data [29]. After dosing 42 mg/Kg *N-*methylbupropion, the AUC values for BupOH, Bup, THB followed a similar profile to dosing of bupropion. THB had the highest AUC at 2.31 μg.h/mL followed by BupOH 1.80 μg.h/mL then Bup 0.64 μg.h/mL. Following administration of bupropion, it’s AUC was approximately twice that after administrating *N-*methylbupropion. This is possibly due to competing metabolism of *N-*methyl-bupropion to the reduced *N-*methylaminoalcohol which was quantified in plasma and brain. In the case of administration of *N-*methylbupropion by intraperitoneal injection, a significant proportion of *N-*methylbupropion must be reduced to the aminoalcohol before *N-*demethylation to bupropion occurs.

Significant amounts of *N-*methylbupropion (*N-*MeBup) were present in the plasma (AUC = 0.23 μg h/mL) and the *N-*methylaminoalcohol (*N-*MeTHB) was also present AUC = 0.13 μg.h/mL. The *C_max_* trend in data reflected the plasma AUCs trend in data but there was a change in the *t_max_* for both THB and BupOH. The longer *t_max_* for THB and BupOH indicated the time spent in conversion from prodrug to bupropion then to metabolites.

The levels of THB, BupOH and Bup in the brain were similar following dosing with the prodrug and bupropion but there was a significant difference in the brain/plasma ratios. When *N-*methyl bupropion was dosed there was an increase in the brain/plasma ratio of bupropion compared to when bupropion was dosed, even though plasma levels were almost half as much. This could be due to *N-*methylbupropion crossing the blood brain barrier and being demethylated *in situ* by brain oxidative enzymes. After prodrug dosing all metabolites including bupropion had a higher brain/plasma ratio.

These results were encouraging, in that the prodrug concept of *N-*methylbupropion had been demonstrated in the guinea pig. *N-*methyl bupropion was successfully converted to bupropion and metabolites *in vivo*. However, significant levels of *N-*methylbupropion and its aminoalcohol metabolite were detected in the plasma and brain. The AUC and *C_max_* of metabolites of bupropion showed significant difference between bupropion and *N-*Methylbupropion when dosed by intraperitoneal injection and were shown by statistical methods not to be the same.

### 3.7. Pharmacokinetics of N-Methyl Bupropion and Bupropion in Guinea Pig Following Oral Administration (PO)

A second PK guinea pig study was performed using oral delivery. Oral delivery involves the full first pass metabolic effect factors, including stability in the stomach, stability in the GI tract and absorption through the intestinal membrane.

**Figure 5:**
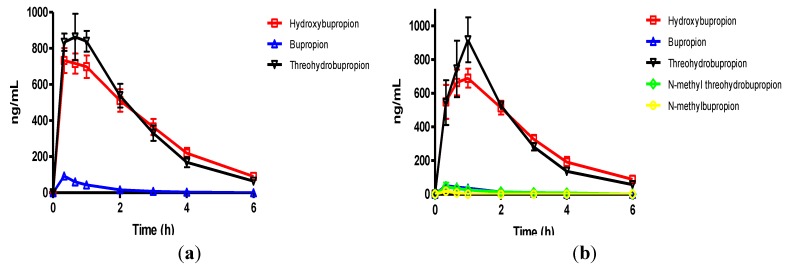
(**a**) The mean plasma concentrations of bupropion and selected metabolites after dosing 40 mg/Kg bupropion to guinea pigs by oral gavage (*n =* 5); (**b**) The mean plasma concentrations of *N*-methylbupropion and selected metabolites after dosing 42 mg/Kg *N*-methylbupropion to guinea pigs by oral gavage (*n =* 5).

**Figure 6:**
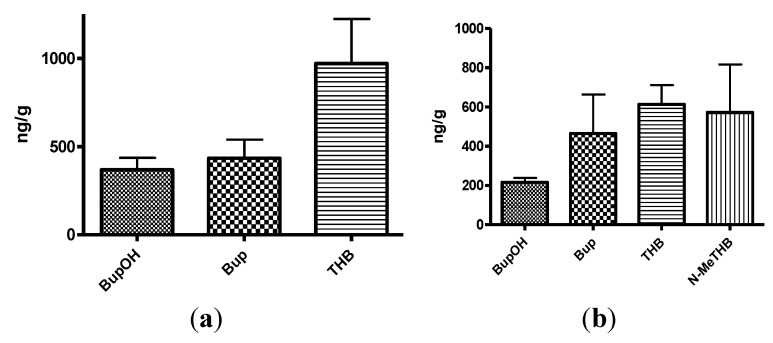
(**a**) Mean brain concentrations of bupropion and metabolites 20 min after dosing 40 mg/Kg bupropion in guinea pig by oral gavage (*n* = 5). Concentrations are expressed in terms of ng of compound per g of brain tissue; (**b**) Mean brain concentrations of *N*-methyl-bupropion and metabolites 20 min after dosing 42 mg/Kg *N*-methylbupropion in guinea pig by oral gavage (*n* = 5). Concentrations are expressed in terms of ng of compound per g of brain tissue.

The dosages of bupropion and *N-*methylbupropion were maintained at 40 mg/Kg and 42 mg/Kg respectively. The sampling time points were increased to six hours post dosing and brain was removed at 20 min to evaluate brain/plasma ratios. Brain levels were determined on a separate group. The pharmacokinetic results are shown in Table 4, Table 5 and Table 6. The results are presented in Figure 5 and Figure 6.

**Table 4:** Estimated pharmacokinetic parameters for N-methylbupropion, bupropion and metabolites following equimolar dosing via oral gavage. Dosed equimolar to bupropion 40 mg/Kg (*n =* 5).

Bupropion dosed at 40 mg/Kg			Parameter	N-Methylbupropion dosed at 42 mg/Kg				
BupOH	Bup	THB	Metabolite	BupOH	Bup	THB	N-Me Bup	N-Me THB
2.25	0.11	2.31	**AUC_0-360 _(µg.h/mL)**	2.10	0.08	2.11	0.08	0.01
0.73 ± 0.15	0.09 ± 0.03	0.83 ± 0.11	**Mean C_max _(µg/mL) ± SD**	0.69 ± 0.13	0.05 ± 0.02	0.92 ± 0.30	0.02 ± 0.01	0.04 ± 0.05
0.33	0.33	0.66	**t_max_ (h)**	1.00	0.33	1.0	0.33	0.33
1.7	0.9	1.3	**t½ (h)**	1.6	0.8	1.1	<LOQ	1.7

**SD** = standard deviation.

**Table 5:** The brain/plasma ratio of bupropion and metabolites in the guinea-pig 20 min post dose of 40 mg/Kg bupropion via oral gavage (*n =* 5).

Metabolite	Brain levels		Plasma levels		Brain/plasma ratio
	μg/g	SD	μg/mL	SD	
**BupOH**	0.37	0.14	0.73	0.15	0.50
**Bup**	0.43	0.21	0.09	0.03	4.78
**THB**	0.97	0.51	0.83	0.11	1.17

**Table 6:** The brain/plasma ratio of *N*-methyl bupropion and metabolites in the guinea-pig 20 min post dose of 42 mg/Kg *N*-methyl bupropion via oral gavage (*n =* 5).

Metabolite	Brain levels		Plasma levels		Brain/plasma ratio
	μg/g	SD	μg/mL	SD	
**BupOH**	0.22	0.04	0.55	0.22	0.40
**Bup**	0.46	0.40	0.05	0.02	9.20
**THB**	0.62	0.19	0.54	0.30	1.15
***N-*MeTHB**	0.57	0.49	0.04	0.05	14.25
***N-*MeBup**	<LOQ	<LOQ	0.02	0.01	<LOQ

The plasma AUC’s for THB, BupOH and Bup were comparable when the prodrug was dosed equimolarly to bupropion. The plasma AUC’s for the metabolites of bupropion, THB and BupOH were statistically similar when dosing bupropion or *N-*methyl bupropion. The *t_max_* for bupropion’s metabolites, THB and BupOH were extended out to 1 h. The plasma *C_max_* values for THB and BupOH were statistically similar between bupropion and *N-*methylbupropion dosing using a 90% confidence interval. Perhaps another important result was that lower levels of *N-*methylbupropion were found in plasma compared to intraperitoneal injection dosing, which suggest the involvement of intestinal enzymes in its metabolism in the oral route.

The amount of *N-*methylbupropion found in the brain was below the limit of quantitation. Either very little of the potential prodrug reached the brain intact or it was metabolized rapidly after crossing the blood brain barrier. The high brain/plasma ratio of bupropion in the brain would suggest that *N-*methyl bupropion crossed the blood brain barrier and was demethylated to some extent *in situ*.

The brain/plasma ratio levels of THB were comparable when bupropion and *N-*methylbupropion were dosed, but there was slightly reduced levels of BupOH following administration of *N-*methyl-bupropion. The *N-*methylaminoalcohol metabolite (*N-*MeTHB) of the potential prodrug was detected in the plasma at low levels but in the brain at high levels, therefore high brain/plasma ratio is seen which might justify further pharmacological assessment of this metabolite. The high levels of this metabolite in the brain would suggest again that *N-*methylbupropion can cross the blood brain barrier followed by reduction *in situ*. The pharmacokinetic data indicate that the profiles of *N-*methyl-bupropion and bupropion are similar, with respect to the key metabolites THB and BupOH.

### 3.8. Tentative Identification of Novel Minor in Vivo Metabolites of N-Methylbupropion

**Figure 7:**
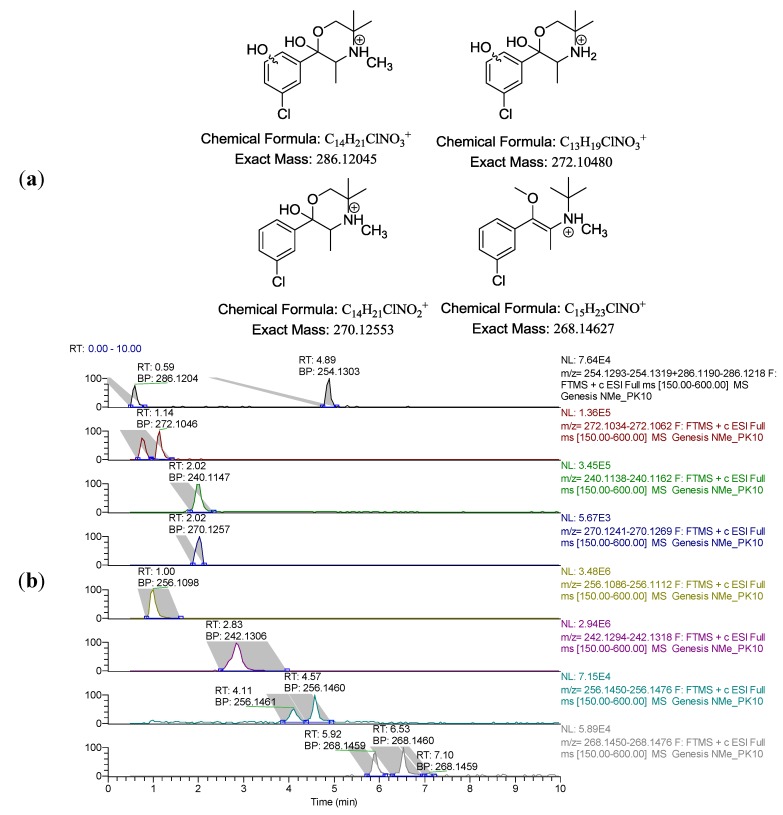
(**a**) Proposed four new metabolites recovered after dosing *N*-methyl-bupropion in both PK guinea-pig studies. Note metabolites are protonated to correlate off MS data in Figure 7(b); (**b**) LC-MS chromatogram of guinea-pig plasma at t = 40 min post prodrug dose. Bupropion (2.02), *threo-*hydrobupropion (2.83), hydroxybupropion (1.00), *N*-methylbupropion (4.89), *N*-methylhydrobupropion isomers (4.11 and 4.57), dihydroxylated metabolite (0.59), demethylated-dihydroxylated metabolite (0.75 and 1.14), monohydroxylated metabolite (2.02), methylated metabolites (5.92 and 6.53).

During both pharmacokinetic studies a number of new metabolites relating to *N-*methylbupropion were identified. These were filtered out from the accurate mass full scans. The mass defect filter on the data dependent acquisitions was set to <5.0 ppm therefore it is highly probable that the molecular formula for these metabolites is correct. The instrument was set in chlorine isotope mode to select out mass ions with a chlorine isotope for data dependent MS/MS. Four main metabolites were tentatively identified with stereoisomers (Figure 7).

The most polar new metabolite was the dihydroxylated metabolite of *N-*methylbupropion (mass 286.1204 amu) at a retention time of 0.6 min. The hydroxylation position could not be assigned as there was a lack of MS^n^ data to say with any certainty the positions of the hydroxyl groups but at least one is likely on the *t*-butyl group, causing cyclisation.

Two diastereomers of the dimethylated-dihydroxylated metabolite were present (mass 272.1048) with retention times of 0.8 and 1.1 min respectively.

The monohydroxylated metabolite of *N-*methylbupropion was present at 2.0 min (mass 270.1255). This monohydroxylated metabolite also has two chiral centres and therefore should exist as two diastereomeric peaks in the chromatogram but only one was present.

A further pair of metabolites were assigned as the *O-*methylated metabolite of *N-*methylbupropion (mass 268.1463), retention time 5.92 and 6.53 min. There were two isomers present, *syn* and *anti*, due to the double bond in the enol group and each of the *syn* and *anti* isomers is chiral. Their identity could be confirmed using authentic standards we had prepared previously as potential prodrugs of bupropion, discussed in paper 1.

## 4. Conclusions

A method was developed and validated for determining *N-*methylbupropion, bupropion and their metabolites in guinea-pig plasma and brain. Administration of bupropion and *N-*methylbupropion in equimolar doses by oral gavage to guinea pig, leads to similar plasma AUC and *C_max_* of bupropion, *threo*-hydrobupropion and hydroxybupropion. Significant differences were observed between IP and oral administration which could be accounted for by intestinal metabolism of *N*-methylbupropion. This compound is a less active analogue of bupropion in DAT and NET assays. It is extensively metabolized to bupropion and its metabolites, and *N-*methyl-*threo*-hydrobupropion *in vivo*. The *t_max_* for the key metabolites hydroxybupropion and *threo*-hydrobupropion following administration of the *N-*methyl compound was extended from 20 to 60 min most likely due to the conversion of *N-*methyl-bupropion to bupropion. The *N-*methyl-*threo*-hydrobupropion metabolite of *N-*methylbupropion is a significant metabolite that is likely to be pharmacologically active.

This data suggests that the *N-*methyl derivative of bupropion may have potential as an oral prodrug of bupropion in humans. The two PK studies indicate the potential for other *N-*alkylated analogues of bupropion to act as bio-precursor prodrugs of bupropion. Finally, the study indicates that the *in vitro* screening tool reported in the paper one of this series may be a generally useful tool in the systematic identification of new prodrugs.

## Supplementary Files

Supplementary File 1 Supplementary Information (DOCX, 166 KB)
