# Supplementary material for: Part Two: Evaluation of N-methylbupropion as a Potential Bupropion Prodrug
Source: Pharmaceuticals (Basel). 2014 May 28;7(6):676–94. doi: 10.3390/ph7060676 (PMC4078515; doi:10.3390/ph7060676)
Supplement: Supplementary File 1 — Supplementary Information (DOCX, 166 KB) [file pharmaceuticals-07-00676-s001.docx]

**Supplemental Information**

**1. ^1^H-NMR and ^13^C-NMR spectrograms of *N*-methyl-*erythro*- and *N*-methyl-*threo*-hydrobupropion standard isomer mixture and calculation of isomeric ratio by ^1^H-NMR.**


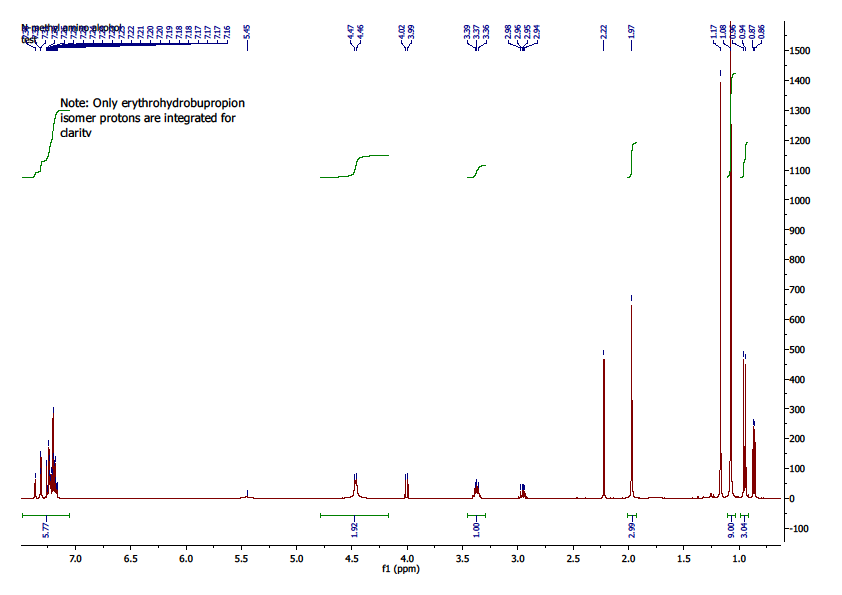


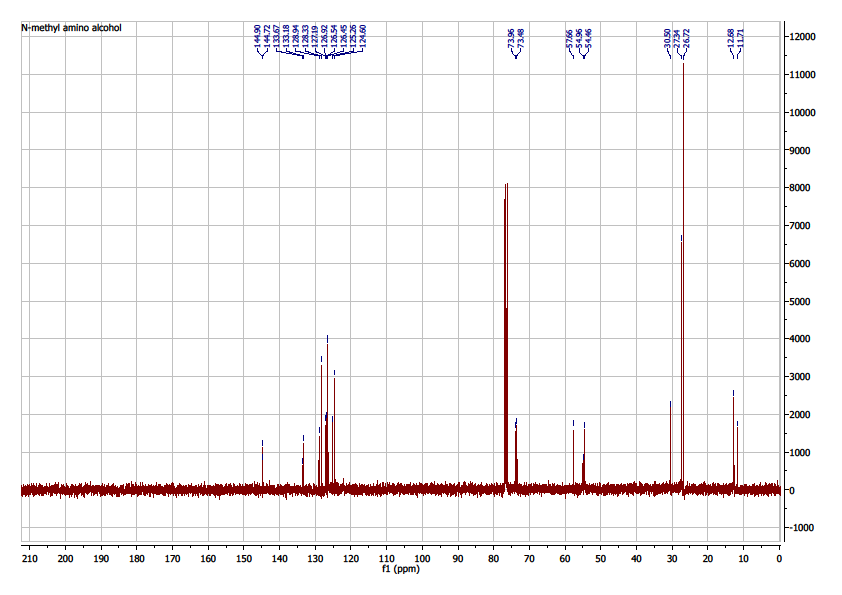

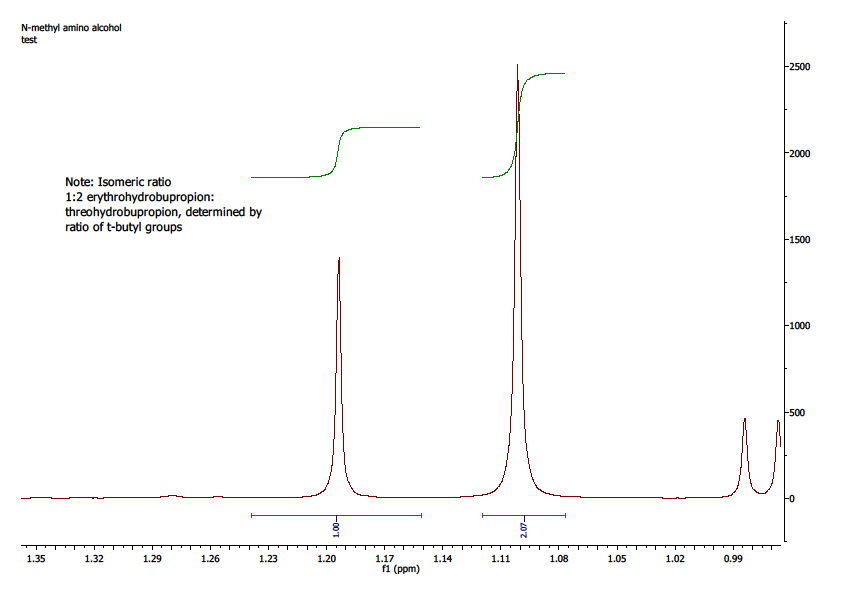


2. Experimental for Analytical Method Validation

2.1. Selectivity

Selectivity solutions were evaluated by preparing all standards, blanks, plasma and brain homogenates separately. The solutions were prepared and diluted appropriately as per method and chromatographed. Minimum resolution, interference and carryover were evaluated.

2.2. Linearity

Stock standard solutions were prepared in methanol on the day of analysis. Standards were prepared by serial dilution of stock standard solutions using 60:40 0.001 M HCl-ACN as diluent. Standards were prepared in the concentration range of 0.1–1000 ng/mL. The linear range was calculated by least squares linear regression analysis. Linear portions of each calibration curve had a correlation coefficient of >0.995.

2.3. Accuracy/Precision

Human plasma was spiked with *N-*methyl bupropion, bupropion and metabolites at three concentrations, 100, 500 and 1000 ng/mL. Aliquots (6 × 50 µL) were transferred to 1.5 mL microcentrifuge tubes. ACN (200 µL) was added and the solution was vortex mixed. The remaining mixture was centrifuged at 10,000 rpm for 10 min. Supernatant (100 µL) was removed and added to 0.001 M HCl (100 µL). The solution was vortex mixed again and an aliquot (100 µL) was placed in a micro-insert for HPLC analysis. The final concentrations were 10, 50, 100 ng/mL.

2.4. Plasma LLOQ/precision

Human plasma was spiked with *N-*methylbupropion, bupropion and metabolites at a concentration of 10 ng/mL. Aliquots (6 × 50 µL) were transferred to 1.5 mL microcentrifuge tubes. ACN (200 µL) was added and the solution was vortex mixed. The remaining mixture was centrifuged at 10,000 rpm for 10 min. An aliquot (100 µL) of supernatant was removed and added to 0.001 M HCl (100 µL).
The solution was vortex mixed again and 100 µL was placed in a micro-insert for HPLC analysis.
The final concentration was 1 ng/mL. All LLOQ solutions had an analyte peak signal to noise ratio greater than 10.

2.5. Plasma LOD

The limit of detection of the method was determined by injection of serially diluted standards from highest to lowest level. The limit of detection was determined as the lowest peak seen with a minimum signal to noise ratio of three.

2.6. Brain LLOQ Accuracy/Precision

Rat brain homogenate (165 mg brain per mL 0.01M HCl) was spiked with *N-*methylbupropion, bupropion and metabolites at two concentrations, 250 and 2500 ng/mL. Aliquots (5 × 200 µL) were transferred to 1.5 mL microcentrifuge tubes. The mixture was centrifuged at 15,000 rpm for 10 min. Supernatant (100 µL) was transferred to 1.5 mL microcentrifuge tubes. ACN (300 µL) was added and the solution was vortex mixed. The remaining mixture was centrifuged at 15,000 rpm for 10 min.
200 µL of supernatant was removed and added to 800 µL of water. The solution was vortex mixed again and analysed by HPLC. Final concentrations were 12.5, 125 ng/mL.

2.7. Pharmacological screening of N-methylbupropion and bupropion

The pharmacological screens were carried out by MDS Pharma Services Taiwan. The methods used are detailed in the following references, NET [[1]](#_ENREF_22), DAT [2,3], Muscarinic acetylcholine receptor [[4]](#_ENREF_25), nicotinic acetylcholine receptor [5,6] and SERT [7,8]. The specific nicotinic acetylcholine assays are detailed in the following references, nicotinic acetylcholine α1 [[9]](#_ENREF_30), nicotinic acetylcholine α4β2 [10,11] and nicotinic acetylcholine α7 [[12]](#_ENREF_33).

3. Results Method Validation

3.1. Specificity

The bioanalytical LCMS method was shown to be specific by injection of all standards, plasma and diluent solutions separately. No interference was observed with mass values similar to the analytes of interest. The ability of the Orbitrap to extract ions ± 2.0 ppm routinely during the course of the analysis significantly improved the signal to noise ratio affording excellent specificity and sensitivity. The diastereomeric pair *rac*-*erythro*-hydrobupropion and *rac-threo*-hydrobupropion which have identical mass (242.1306 amu) required baseline resolution for accurate quantitation. Resolution between these two compounds over the linear range was >1.5 which was considered acceptable. The same resolution was observed for the diastereomeric pair of *N-*methylaminoalcohol metabolites. For use of this method for determination of bupropion and metabolites in guinea-pig plasma and brain, selectivity is not an issue for *rac-erythro*-hydrobupropion as this is not generated in guinea pigs in significant amount [[13]](#_ENREF_3).

3.2. Linearity

Linearity was assessed by injection of a range of standards from 0.1 ng/mL to 1000 ng/mL. The linear part of the calibration curve was evaluated by least squares-linear regression analysis where the linear range was calculated by the range of points with a correlation coefficient of greater than 0.995. The method was found to be linear for all analytes for at least three orders of magnitude, with a minimum correlation coefficient of r^2^ ≥ 0.995. Table S1 presents the correlation coefficient values for bupropion, *N-*methylbupropion and their metabolites. The linear range for *rac*-*N-*methylaminoalcohols was lower as this standard was 2:1 mixture of the two diastereomers. *N-*methylbupropion had the largest range of quantitation, 1–500 ng/mL.

**Table S1.** Shows correlation levels for bupropion and its principal human metabolites in the range 1–250 ng/mL.

| **Standard** | **Linear Range (ng/mL)** | **r^2^** |
| --- | --- | --- |
| Hydroxybupropion | 1–250 | 0.9984 |
| *Rac-erythro*-hydrobupropion | 1–250 | 0.9951 |
| *Rac-threo*-hydrobupropion | 1–250 | 0.9970 |
| *Rac*-*N-*methyl-*erythro*-hydrobupropion | 1–165 | 0.9989 |
| *Rac-N-*methyl-*threo*-hydrobupropion | 1–85 | 0.9995 |
| *N-*methylbupropion | 1–500 | 0.9996 |
| Bupropion | 1–250 | 0.9991 |

3.3. Carryover

Carryover was assessed by injection of a blank sample following analysis of the highest concentration linearity sample. Initially during the method development there was carryover (>2%) of bupropion but after careful adjustment of needle flushing and washing between injections, this was eliminated. The needle wash solution was optimized as 0.5% formic acid in MeOH. The acidified needle wash improved system suitability, reproducibility and eliminated carryover (<0.1%).

3.4. Limit of detection

The limit of detection was determined as the lowest peak detected with a minimum signal to noise ratio of three. The LOD of *N-*methylbupropion, bupropion and metabolites was shown to be in the pg/mL range (Table S2).

**Table S2.** Signal to noise ratios (S/N) at the nominal LOD levels.

| **Standard** | **LOD (ng/mL)** | **S/N** |
| --- | --- | --- |
| Hydroxybupropion | 0.25 | 4 |
| *Erythro-*hydrobupropion | 0.25 | 3 |
| *Threo-*hydrobupropion | 0.25 | 3 |
| *N-*Methylbupropion | 0.25 | 4 |
| Bupropion | 0.50 | 5 |

3.5. Accuracy and precision

The accuracy/recovery and precision of the method was evaluated using human plasma. Six replicate plasma samples were analysed at three different levels across the linear range. Each was spiked with *N-*methylbupropion, bupropion and metabolites in the expected pharmacokinetic concentration range. All accuracy results were within 85%–115%, % RSD ≤ 15% (Table S3).

**Table S3.** Accuracy/precision results for hydroxybupropion, *threo*-hydrobupropion, bupropion and *N*-methylbupropion.

| **Theoretical Conc. Level (ng/mL)** | **Actual Conc. Level (ng/mL)** | **± (SD)** | **%RSD** | **Accuracy (%)** |
| --- | --- | --- | --- | --- |
| **Hydroxybupropion** |  |  |  |  |
| 10.00 | 10.23 | 0.35 | 3.43 | 102.33 |
| 50.00 | 53.04 | 1.67 | 3.16 | 106.07 |
| 100.00 | 100.64 | 4.70 | 4.67 | 100.64 |
| **Mean** |  |  | **3.75** | **103.01** |
| ***Threo*-hydrobupropion** |  |  |  |  |
| 10.00 | 11.02 | 0.40 | 3.60 | 110.25 |
| 50.00 | 59.22 | 2.08 | 3.62 | 118.44 |
| 100.00 | 109.39 | 3.96 | 3.62 | 109.39 |
| **Mean** |  |  | **3.61** | **112.69** |
| **Bupropion** |  |  |  |  |
| 10.00 | 9.03 | 0.58 | 6.41 | 90.33 |
| 50.00 | 51.34 | 1.92 | 3.74 | 102.67 |
| 100.00 | 97.77 | 3.73 | 3.82 | 97.77 |
| **Mean** |  |  | **4.65** | **96.92** |
| ***N-*Methylbupropion** |  |  |  |  |
| 10.00 | 9.33 | 0.56 | 5.95 | 93.31 |
| 50.00 | 57.61 | 2.75 | 4.77 | 115.21 |
| 100.00 | 113.79 | 4.38 | 3.85 | 113.79 |
| **Mean** |  |  | **4.86** | **107.44** |

3.6. LLOQ-Accuracy and precision

Plasma lower limit of quantitation-accuracy and precision was established at the 1 ng/mL level for *N-*methylbupropion, bupropion and metabolites. At 1 ng/mL each analyte had a signal to noise ratio of greater than 10. The results are tabulated in Table S4.

**Table S4.** Plasma LOQ accuracy and precision results.

| **Standard** | **Theoretical Conc. Level (ng/mL)** | **Actual Conc. Level (ng/mL)** | **± (SD)** | **%RSD** | **Accuracy (%)** |
| --- | --- | --- | --- | --- | --- |
| **Hydroxybupropion** | 1.0 | 1.09 | 0.05 | 4.90 | 108.97 |
| ***Threo*-hydrobupropion** | 1.0 | 0.96 | 0.23 | 24.37 | 95.88 |
| ***N-*Methylbupropion** | 1.0 | 1.18 | 0.24 | 20.28 | 118.34 |
| **Bupropion** | 1.0 | 1.18 | 0.12 | 10.19 | 118.14 |

Brain LLOQ was determined at 12.5 ng/mL and accuracy/recovery at 125 ng/mL. The CV of six replicates at each level was higher than that of plasma samples. This was attributed to dissolved protein precipitating during sample preparation. This displaces the solution volume and can have an effect of concentrating the solutions. Results are tabulated in Table S5.

**Table S5.** Brain LOQ/accuracy and precision results.

| **Compound** | **Level (ng/mL)** | **Mean (ng/mL)** | **SD** | **%RSD** | **Accuracy (%)** |
| --- | --- | --- | --- | --- | --- |
| **Hydroxybupropion** | 12.5 | 14.87 | 1.00 | 6.75 | 118.93 |
|  | 125 | 132.71 | 4.35 | 3.28 | 106.17 |
| ***Threo-*hydrobupropion** | 12.5 | 14.98 | 0.80 | 5.37 | 119.87 |
|  | 125 | 137.30 | 5.01 | 3.65 | 109.84 |
| **Bupropion** | 12.5 | 16.50 | 1.58 | 9.56 | 131.98 |
|  | 125 | 145.59 | 6.90 | 4.74 | 116.47 |
| ***N-*Methylated bupropion** | 12.5 | 15.10 | 0.64 | 4.27 | 120.84 |
|  | 125 | 166.10 | 5.04 | 3.03 | 132.88 |

3.7. Sample and standard stability

Sample and standard stability was evaluated over 24 h. Samples and standards were shown to
be >90% assay after 24 h when stored at 5 °C. Medium and long term stability was not established. Sample and standard stability in plasma is already documented in literature for bupropion and metabolites [[14,15]](#_ENREF_8). Bupropion plasma samples are stable at low pH and metabolite samples are stable in the pH range 2.5–10.0. Therefore our samples and standards were prepared in 0.001 M HCl. This is the standard sample preparation procedure in the current USP monograph for preparation of assay samples for bupropion hydrochloride extended release tablets [[16]](#_ENREF_10). Standard stability was demonstrated throughout the run and at the end of the run by calculating the%RSD of a repeat injection standard. This verified standard stability over the course of the analysis.

References

1. Galli, A.; DeFelice, L.J.; Duke, B.J.; Moore, K.R.; Blakely, R.D. Sodium-dependent norepinephrine-induced currents in norepinephrine-transporter-transfected hek-293 cells blocked by cocaine and antidepressants. *J. Exp. Biol.* **1995**, *198*, 2197–2212.

2. Giros, B.; Caron, M.G. Molecular characterization of the dopamine transporter. *Trends Pharmacol. Sci.* **1993**, *14*, 43–49.

3. Gu, H.; Wall, S.C.; Rudnick, G. Stable expression of biogenic amine transporters reveals differences in inhibitor sensitivity, kinetics, and ion dependence. *J. Biol. Chem.* **1994**, *269*,
7124–7130.

4. Luthin, G.R.; Wolfe, B.B. Comparison of [3h]pirenzepine and [3h]quinuclidinylbenzilate binding to muscarinic cholinergic receptors in rat brain. *J. Pharmacol. Exp. Ther.* **1984**, *228*, 648–655.

5. Davila-Garcia, M.I.; Musachio, J.L.; Perry, D.C.; Xiao, Y.; Horti, A.; London, E.D.;
Dannals, R.F.; Kellar, K.J. [125i]iph, an epibatidine analog, binds with high affinity to neuronal nicotinic cholinergic receptors. *J. Pharmacol. Exp. Ther.* **1997**, *282*, 445–451.

6. Whiteaker, P.; Jimenez, M.; McIntosh, J.M.; Collins, A.C.; Marks, M.J. Identification of a novel nicotinic binding site in mouse brain using [(125)i]-epibatidine. *Br. J. Pharmacol.* **2000**, *131*, 729–739.

7. Shearman, L.P.; McReynolds, A.M.; Zhou, F.C.; Meyer, J.S. Relationship between
[125i]rti-55-labeled cocaine binding sites and the serotonin transporter in rat placenta. *Am. J. Physiol.* **1998**, *275*, C1621–C1629.

8. Wolf, W.A.; Kuhn, D.M. Role of essential sulfhydryl groups in drug interactions at the neuronal 5-ht transporter. Differences between amphetamines and 5-ht uptake inhibitors. *J. Biol. Chem.* **1992**, *267*, 20820–20825.

9. Davies, A.R.; Hardick, D.J.; Blagbrough, I.S.; Potter, B.V.; Wolstenholme, A.J.; Wonnacott, S. Characterisation of the binding of [3h]methyllycaconitine: A new radioligand for labelling alpha 7-type neuronal nicotinic acetylcholine receptors. *Neuropharmacology* **1999**, *38*, 679–690.

10. Lukas, R.J. Characterization of curaremimetic neurotoxin binding sites on membrane fractions derived from the human medulloblastoma clonal line, te671. *J. Neurochem.* **1986**, *46*, 1936–1941.

11. Sine, S.M. Functional properties of human skeletal muscle acetylcholine receptors expressed by the te671 cell line. *J. Biol. Chem.* **1988**, *263*, 18052–18062.

12. Pabreza, L.A.; Dhawan, S.; Kellar, K.J. [3h]cytisine binding to nicotinic cholinergic receptors in brain. *Mol. Pharmacol.* **1991**, *39*, 9–12.

13. Suckow, R.F.; Smith, T.M.; Perumal, A.S.; Cooper, T.B. Pharmacokinetics of bupropion and metabolites in plasma and brain of rats, mice, and guinea pigs. *Drug Metab. Dispos.* **1986**, *14*, 692–697.

14. Laizure, S.C.; DeVane, C.L. Stability of bupropion and its major metabolites in human plasma. *Ther. Drug Monit.* **1985**, *7*, 447–450.

15. Cooper, T.B.; Suckow, R.F.; Glassman, A. Determination of bupropion and its major basic metabolites in plasma by liquid chromatography with dual-wavelength ultraviolet detection.
*J. Pharm. Sci.* **1984**, *73*, 1104–1107.

16. Ravichandran, R. Usp monographs: Bupropion hydrochloride extended-release tablets.
Available online: http://www.pharmacopeia.cn/v29240/usp29nf24s0_m10524.html (accessed on 30 April 2014).

© 2014 by the authors; licensee MDPI, Basel, Switzerland. This article is an open access article distributed under the terms and conditions of the Creative Commons Attribution license (http://creativecommons.org/licenses/by/3.0/).
